# Designing effective virtual reality environments for pain management in burn-injured patients

**DOI:** 10.1007/s10055-021-00552-z

**Published:** 2021-06-22

**Authors:** Ivan Phelan, Penny J Furness, Maria Matsangidou, Nathan T. Babiker, Orla Fehily, Andrew Thompson, Alicia Carrion-Plaza, Shirley A. Lindley

**Affiliations:** 1 Centre for Culture, Media and Society, College of Social Sciences and Arts, Sheffield Hallam University, Sheffield, S1 1WB UK; 2 Centre for Behavioural Science and Applied Psychology (CeBSAP), College of Social Sciences and Arts, Sheffield Hallam University, Sheffield, S1 1WB UK; 3 Department of Psychological Services, Sheffield Teaching Hospitals NHS Foundation Trust, Sheffield, S10 2JF UK; 4 Department of Psychology, University of Sheffield, Sheffield, S1 2LT UK

**Keywords:** Virtual reality, Burn injuries, Pain, Anxiety, Interactivity, Patient-centred design

## Abstract

Burn patients engage in repetitive painful therapeutic treatments, such as wound debridement, dressing changes, and other medical processes high in procedural pain. Pharmacological analgesics have been used for managing pain, but with ineffective results and negative side effects. Studies on pain management for burn patients suggested that Virtual Reality can treat procedural pain. This paper describes the process of designing, testing, and deploying a Virtual Reality system into a hospital setting. Firstly, a workshop was conducted to identify the most suitable types of Virtual Reality contents for the needs of burn-injured patients. Then, an experimental study, with 15 healthy adults, explored the analgesic impact of the Virtual Reality contents. The pain was induced through a cold pressor. Finally, we deployed the Virtual Reality system into the hospital to examine its efficiency on burn-injured inpatients. This study presents factors for the effective design and deployment of Virtual Reality for burn-injured patients residing in a hospital. Those factors refer to the use of cartoonish features and a choice of content based on each patient’s interests to increase the positive emotions and the use of interactive features, portable equipment to reduce pain and increase the feasibility of the technology in clinical settings. Finally, our results indicated that the extension of the VR use after the therapeutic session could support more effective pain treatment.

*Trial registration number* Protocol ID: AA8434.

## Introduction

Burn-injured patients’ rehabilitation requires dealing with painful therapeutic processes. Although these processes are fundamental for their recovery, by improving the functional outcomes and minimizing persistent disabilities, burn-injured patients often neglect to participate fully in their therapies (Richardson and Mustard 2009) due to the significant procedural pain (Ehde et al. 1998; Patterson and Sharar 2001). This has led to the emergence of research for providing feasible solutions which aim to support the burn-injured patients’ therapeutic processes since promoting and enhancing the therapeutic processes of burn-injured patients can be considered a model measure of effective patient care.

Research has shown that burn-injured patients are usually dealing with a greater sensitivity to infection and acute stress symptoms (Stoddard et al. 2006), post-traumatic stress disorder, concerns about the impact on appearance (Berger et al. 2010), suicide post-discharge (Macleod et al. 2016; Mahar et al. 2012) and loss of confidence in the care team (Edwards 2011). As a result, further research is needed to explore and develop novel interventions that can support and enhance the burn-injured patients’ painful therapeutic processes while receiving care within such potentially restricted environments.

This study aimed to understand how virtual reality (VR) can be deployed into a hospital environment based on the restrictions such technology possesses and how to design effective Virtual Environments for burn-injured patients. The study aimed to contribute to research in the design community by presenting a long-term experimental study for the effective design of a more deployable VR system for burn-injured patients dealing with painful therapeutic processes within hospital environments.

### Burn-injured patients care

Most of the burn-injured patients undergo painful repetitive therapeutic processes, such as wound debridement, dressing changes, wound cleaning, limb mobility exercises and therapeutic skin stretching. The perception of pain associated with burn injuries has been reported as one of the most intense types of pain. Therefore, the pain that accompanies the burn injuries treatment presents an important challenge, met not only to the patients but also to the clinical staff (Patterson et al. 2004).

A variety of pharmacological analgesics have been used for the treatment of burn injury pain (Patterson and Sharar 2001), with unwanted side effects and ineffective results reported by several patients (Carrougher and Patterson 2002; Choiniere et al. 1992; Ohrbach et al. 1998; Perry et al. 1981; Ptacek et al. 1995). Research has shown that pharmacological analgesics may only provide effective pain relief to 25% of burn-injured patients (Miller et al. 1992). Research has also demonstrated some side effects associated with the constant intake of pharmacological analgesics, such as addiction, nausea, constipation, sedation, itchiness, urinary retention, cognitive impairment, hallucinations, and respiratory depression (Brown et al. 2000; Cherny et al. 2001). It is therefore important to find non-pharmacological interventions for managing burn injury pain.

Many studies on burn care suggest that psychological interventions in concert with pharmacological analgesics can improve rehabilitation and reduce procedural pain (Patterson and Ptacek 1997). Psychological interventions have also been shown to reduce (Lang et al. 2000; Wakeman and Kaplan 1978) or even eliminate (Finer and Nylen 1961; Ohrbach et al. 1998) the need for pharmacological analgesics.

The most well-known psychological intervention for the treatment of pain in burn-injured patients is distraction. Distraction was found to be able to reduce the perception of procedural pain by the subject during the therapy. Research has shown effective results in pain management based on distraction via imagery, meditation, relaxation, hypnosis, and positive thinking (Bernstein 1965; Patterson and Ptacek 1997; Patterson et al. 1987; Patterson and Sharar 2001). Examples of distraction techniques include deep breathing, video viewing, bubble blowing, reading stories, listening to music or singing (Cassidy et al. 2002; De Jong 2013; Miller et al. 1992; Seers and Carroll 1998). The effectiveness of distraction falls within the scope of cognitive-behavioural therapy found in 47 meta-analysis studies to reduce pain episodes by up to 85% (Fernandez and Turk 1989).

### Virtual reality burn-injured patients care

Many studies on burn care (Carrougher et al. 2009; Furness et al. 2019; Hoffman et al. 2014; Kipping et al. 2012; Maani et al. 2011; Schmitt et al. 2011) and induced pain via thermal stimuli (Czub and Piskorz 2012; Hoffman et al. 2004, 2007; Phelan et al. 2019), suggested that VR can be a suitable solution for procedural pain management. This is because VR allows the users to experience a computer-simulated reality based on visual cues and enhanced with auditory and, in due course, tactile and olfactory interactions. Therefore, VR provides the user with a complete distractive illusion of different senses (Li et al. 2011).

A systematic literature review on VR for pain management found that all the VR burn care studies carried out since 2009 employed distraction designed to manage procedural burn pain (Matsangidou et al. 2017a, b). The study suggested that two types of VR-distraction are used from the general bibliography for pain management in burn-injured patients: (a) Single and (b) Advanced Distraction.

To illustrate that, the single type of distraction requires the patient to engage in an immersive experience of playing a VR game, so as to be distracted from the painful signal produced by the burn care process. For example, burn-injured patients were asked to play a software game based on the appropriate age limit during burn wound care (Kipping et al. 2012). The advanced type of distraction requires the patient to play a VR game enhanced by ice-features. For instance, burn-injured patients were asked to play a software game which was taking place in a snowy environment with ice-features (Carrougher et al. 2009; Hoffman et al. 2014; Maani et al. 2011; Markus et al. 2009; Schmitt et al. 2011). Snow-Virtual Environments can create an illusion of a “cooling” effect, and research findings have suggested a strong link between viewing cool environments perceiving reduced pain in the burn-injured population.

Research has shown that colours are also highly relevant to human visual perception by endorsing associations with temperature. For example, red is usually associated with “heat” while blue is mostly related to “cold” (Moseley and Arntz 2007). Several studies have supported an association between colours and thermal perception with blue colour to be highly correlated with cooling sensations and red with burdening (Candas and Dufour 2005; Durgin et al. 2007). To further corroborate the above, studies have shown that individuals' pain tolerance is increased when a stimulus is linked to a blue visual cue than when the same stimulus is linked to a red visual cue (Moseley and Arntz 2007; Martini et al. 2013).

Given the growing evidence for the VR effectiveness on burn pain, the limited side effects (Garrett et al. 2014) and the possibilities such technology offers [e.g. (a) immersing the patient into a “cold” virtual environment to distract him/her from perceiving nociceptive signals and pain; and (b) alter the colours of the VR environment to promote cooling sensations], reviewers have recommended the deployment of VR in clinical settings (Schneider 2017). However, only a few studies have examined how to effectively deploy the system in a hospital setting and how to design software to meet the subjective needs of this particular patient group (Bucolo et al. 2006; Le May et al. 2016).

This study aims to understand how VR can be deployed into a hospital environment based on the restrictions such technology possesses and how to design effective Virtual Environments for burn-injured patients. We believe that there is a lot to be learnt regarding the potential of deploying VR technology within more complicated healthcare settings, such as hospitals, where the types of burn injuries and the patients’ interests may vary. As a result, these challenges increase the need for designing meaningful and effective VR environments that can be tailored to the specific needs of different burn-injured patient groups.

## Methods

### Study design and procedure

The study was based on three phases. During Phase 1, a workshop was conducted to discuss potential VR environments focussed on identifying suitable types of VR content for the needs of burn-injured patients and define the aspects that must be avoided. The workshop was run with professionals in games development and psychologists with expertise in burn care. The findings of Phase 1 were taken into consideration to develop virtual environments.

In Phase 2, we ran an experimental study aimed to explore the suitability of four VR contents and system based on the experiences reported by the users. Acceptability of the four VR contents and the analgesic impact the VR might have on healthy adults when pain was induced via a cold pressor were tested. The system evaluation phase took place on the University premises. The experiment required the participant to pay one 1-h visit to the laboratory. During the visit, a VR familiarization session took place, with an unrelated to the study virtual environment presented to the user to ensure that the participant could use VR without any side effects. During the experiment, the non-dominant hand of the participant was placed into the cold pressor for five minutes without the use of the VR headset. Then, four different virtual environments were presented to the participant on a counterbalanced design to reduce the risk of carry-over effects, while the participant’s non-dominant hand was placed into the cold pressor. The participant was able to terminate the session once the induced pain was considered to be unbearable. After each session, the participant’s hand was removed from the cold pressor to regain the normal temperature. For health and safety reasons, the maximum exposure time was set to five minutes for each virtual environment. The findings of Phase 2 informed us about the suitability of the VR contents and contributed to the selection of the final virtual environments that were deployed into the hospital settings in Phase 3.

Phase 3 deployed the system into the hospital setting and examined the efficiency, practicality, acceptability and analgesic impact of the system by burn-injured patients. Burn-injured patients were invited to use VR with their usual clinician in a familiar room of the hospital. Burn-injured patients took part in three wound dressings (involving dressing removal, wound cleaning and debridement, and application of fresh dressings). One was conducted without VR and one with VR, in a countered-balanced design. Decisions about the suitable timing of each were made between the patient, the clinical team and the researcher. Prior to the experiment, a familiarization session took place to ensure that the patient could use VR without any side effects.

### Ethics

Phase 1 and Phase 2 were approved by the University’s Research Ethics Committee (PHE-298 and 328-FUR). Phase 3 was approved by the Patient and Public Involvement (PPI) Panel, Directorate of Therapeutics and Palliative Care, Sheffield Teaching Hospitals National Health Service (NHS) Foundation Trust, and NHS Research Ethics Committee (IRAS: 221071). Before giving written consent, participants were fully informed about the study, tried out a short VR experience and had the opportunity to ask questions. The study was performed in accordance with the Declaration of Helsinki.

### Participants

*Phase 1* Seven professionals in game development (*n* = 1), psychology with expertise in burn care (*n* = 1), academia (*n* = 2) and nursing (*n* = 1) along with burn survivors (*n* = 2) participated in this the study. Participants were males = 3 and females = 4, aged between 25 and 65 years (*M* = 41.71, SD = 13.23).

*Phase 2* Fifteen healthy participants (males = 10 and females = 5) aged between 18 and 49 years (*M* = 25.53, SD = 9.55) participated in this the study. All participants had normal or corrected to normal vision and no pre-existing painful conditions, such as fibromyalgia, sports or hand injuries, that could affect their perception of pain induced by the cold pressor. All participants had no history of mental health disorders, migraines, or nausea.

*Phase 3* Five inpatients at the Hospital’s Local Burns Unit who were undergoing regular dressing changes during the study period (males = 2 and females = 3) aged between 19 and 68 years (*M* = 48.2, SD = 19.68) participated to the study. Three out of five participants had flash burns on their hands, arms, and legs, and two out of five participants had scald burns on their legs, abdomen, and thighs. The percentage of flash burns was between 18–20%, and the percentage of scald burns was between 3–4%. Overall, the dressing changes time was between 12 min to 1-h and 10 min (*M* = 36.2, SD = 22.1). Patients had none of the exclusion criteria: head and/or neck burns, wound infection, a current diagnosis of PTSD, active psychotic symptoms, or high levels of distress that could possess a risk for their well-being.

### Materials

#### Phase 1

*Observation Notes* were taken based on the workshop discussions. These observations aimed to identify potential VR environments and activities focussed on the patients’ needs.

#### Phase 2

Semi-structured interviews were conducted with each participant by a clinical researcher after each virtual environment. The aim of these interviews was to gather comments regarding the experience including the following: virtual environment preference, enjoyment, objects appearance, immersion in the virtual environments, and perceived impact on pain.

Quantitative measurements were also collected to assess the VR suitability: Pain Occurrence was measured in seconds and was the first point at which pain was reported by the patient. Pain tolerance was measured in seconds and was the duration before the pain became unbearable and the patient decided to end the session. Preference was measured using ordinal inputs by the user. Each user had to arrange the virtual environment in order based on his/her preference (1 = most liked and 4 = less liked).

#### Phase 3

Observation notes were taken by an HCI researcher to classify the interactions and behavioural responses towards the VR experience. This was done to identify the design and deployment issues, which can help inform the VR design.

Semi-structured interviews were conducted with each patient and clinical staff to identify their experience of each dressing change. Interviews contributed to increasing our awareness about the deployment challenges of the VR system in hospitals, where environmental and procedural restrictions exist.

Quantitative measurements were also collected to assess the VR suitability in clinical settings for burn-injured patients: Pain was measured on a scale range 0–100 during (which referred to the level of pain the patient was feeling during the dressing changes) and after (the level of pain the patient experienced right after the dressing changes, two hours and four hours after the dressing changes). Anxiety was measured in on a scale range 0–100 during the dressing changes.

### Data analysis

*Phase 1* Observational notes were unpacked, and design principles were identified for the design of the virtual environments.

*Phase 2* To explore the suitability of the system based on the user experience and analgesic impact of VR in healthy adults where pain was induced via a cold pressor, descriptive statistics along with content analysis on the interviews data were used.

*Phase 3* To explore the suitability of the VR system, descriptive statistics were used. To understand the system’s deployment challenges and outline the solutions, content analysis was conducted.

### Apparatus

*Phase 2* A cold pressor using an iced water tank, with water circulated to maintain a temperature of 4 °C was used to induce experimental pain to the subject. A digital thermometer with a calibration certificate was used to monitor the temperature. The subject was instructed to place the non-dominant hand into the iced water tank as the dominant hand was required to control the interactive virtual environments. It is noted that cold pressor has been used in several studies to induce pain to the subject as this temperature was found to provide an uncomfortable experience without causing tissue damage (Dahlquist et al. 2008; Law et al. 2010; Sil et al. 2014).

*Phase 1–2–3* An Oculus Rift VR head mounted display (HMD) system was used to stream the audial and visual content. An Oculus Rift remote along with Oculus head tracing was used as interactivity device to allow the user to navigate and interact with the virtual environments. The VR system was developed using the Unity 5.6 and Oculus SDK. The 3D models were sourced from the Unity asset store.

The VR content was displayed on a laptop screen, mirroring the user’s real-time virtual interactions. Open Broadcaster Software 23.0.1 was used as a video screen recorder to record the virtual sessions, the interactions and the discussion between the user and the researcher. A digital recorder was used when interviewing the participants.

## Results and discussion

### Phase 1: virtual environments selection process

A 120-min consultative workshop was conducted at a Burns Care Conference organized by the University. Attendees were a group of seven burn care specialists such as clinical psychologists, nurses and supervisors within burn healthcare. During the workshop, a game development researcher demonstrated the possibilities of VR technology using an Oculus Rift device. Afterwards, attendees suggested suitable VR content for the needs of burn-injured patients. Attendees suggested the following categories: (1) Entertainment (e.g. shows, short videos); (2) Nature (e.g. beach, forest); (3) Calming Experiences (e.g. calm music, regular rhythm); (4) Ice-Cold Environments (e.g. winter snowy forest, frozen ocean); (5) Emotions (e.g. empathic videos were the patients will be able to connect their inner feelings with); (6) Funny Videos (e.g. humans laughing out loud); and (7) Hobbies and Sports (e.g. gardening, basketball). Based on the above categories and the strong clinical background in psychology among the professionals, the following criteria were agreed as avoidance factors: (1) Discordant Experiences (e.g. audial content that is not coherent to the visual feedback); (2) Crowded Environments (e.g. pubs, restaurants); (3) Disturbing Experiences (e.g. loud music, arrhythmic sounds); (4) Warm Environments (e.g. anything relevant to heat such as kettles, bright sun, the red colour); and (5) Emotions (e.g. anything that might upset the patient).

Through the workshop and based on the technical experience of the researchers in the HCI and game development field, the following criteria were agreed in order to appropriately design or select the potential virtual environments: Videos must have: (1) High resolution appropriate to the hospital’s internet connection, to avoid blurry virtual environments; (2) Smooth transitions between scenes to avoid confusion, anxiety and distress; and (3) Stable camera recording to avoid causing motion sickness.

**Fig. 1:**
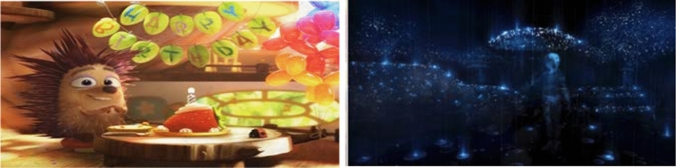
To the Left: A screenshot of the birthday celebration of a Hedgehog. To the Right: A Screenshot of documentary on visual impairments

**Fig. 2:**
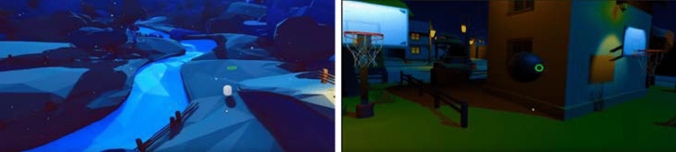
To the Left: A screenshot of the puzzle-based virtual environment. To the Right: A screenshot of the basketball virtual environment

Based on these criteria, four virtual environments were included in the study (see Figs. 1, 2). Two were free to access non-interactive 360° video-based and two were interactive virtual environments. Non-interactive virtual environments were: (1) Birthday celebrations of a hedgehog, and (2) Documentary of a person with visual impairment. Non-interactive virtual environments were selected from a range of videos in each category, based on the ratings the attendees provided using a Likert scale 1–5. Interactive virtual environments were as follows: (1) A puzzle-based virtual environment, and (2) A basketball virtual experience.

#### Virtual environments and rationales

##### Birthday celebration of a hedgehog

*Narrative* The 360° video Henry begins with a cute little hedgehog who has no friends because it likes to hug everyone, but its quills tend to hurt them. The viewer is then placed inside the hedgehog's house on its birthday where, sad and alone, the hedgehog lights up a candle on its birthday cake. The hedgehog then blows the candle out and makes a wish. The wish comes true and a group of animal balloons come to life and fly around the house. The hedgehog happily hugs an animal balloon which pops. Terrified, the animal balloons fly out of the door, leaving the hedgehog alone. Shortly afterwards, the animal balloons return with a turtle. The hedgehog hugs the turtle, which does not get hurt by his quills, and they live happily ever after. The 360° video is a production of Oculus Story Studio, created in Unreal Engine 4. The 360° video has won an Emmy Award for 'Best outstanding original interactive program' (see Fig. 1).

*Rationale* Body image dissatisfaction is highly associated with burn-injured patients with negative consequences for mental health and social life. More importantly, social difficulties, depressive symptoms and the feeling of sadness are associated to body image dissatisfaction which results from burn injuries (Fauerbach et al. 2000, 2002; Thombs et al. 2007, 2008). There is an obvious association between burn-injured patients and the hedgehog since both are facing difficulties in socializing based on their body image. Through the narrative, a positive meaning is given to the patient. Distraction, via cartoonish features, was used to enhance the tolerance to pain (Gold et al. 2006). In the past, cartoons’ distracting nature was found to help in reducing anxiety in clinical environments (Cohen et al. 1997; Lee et al. 2012).

##### Documentary on visual impairments

*Narrative* The film profiles the writer and theologian John M. Hull, who became blind after decades of steadily deteriorating vision. To make sense of the upheaval in his life, Hull began documenting his experiences on audio cassette and wrote his autobiography. Oculus present to the user the audio cassette scenes which are described by Hull in blurry, bluish 360° video scenes. The documentary has won a British Independent Film Award for Best British Documentary (see Fig. 1).

*Rationale* As with the birthday celebrations of a hedgehog, an impairment that produces social difficulties for the person is presented to the patient to enhance empathic reactions. Also, the scenes are presented based on a blue palette related to cooling sensations (Candas and Dufour 2005; Durgin et al. 2007) and pain tolerance (Martini et al. 2013; Moseley and Arntz 2007).

##### Puzzle-based experience

*Narrative* The user was instructed to control a shepherd represented as a white cylinder shape. Using the head tracking which is incorporated into the oculus rift HMD the patient-controlled the movement of the shepherd. During the exposure, the user’s eye gaze location was represented as a green circle in the virtual environment. A single button press of the remote made the shepherd move via an AI navmesh. The aim was to herd the sheep that were represented as different coloured cylinders into their pens by positioning the shepherd to direct the sheep. Obstacles, increased numbers of sheep and restricted areas were presented to the user to increase his/her concentration and attention. All agents (e.g. shepherd and sheep) were given shapes based on an abstract art style. Agents were coupled with sheep sound effects to reinforce the shapes representations (see Fig. 2).

*Rationale* Similarly, to the documentary on visual impairments the virtual environment designed using blue tint to the lighting to induce a cooling effect to the user. In addition, based on the notion that pain perception is affected by the level of attention the individual pays to the sensory signal of the pain (Gold et al. 2007; Melzack and Wall 1965), we designed a game that was able to withdraw the user’s attention from the painful sensory signal by placing it on the task performance.

##### Basketball experience

*Narrative* The users found themselves in a virtual housing basketball estate. They were instructed to look at the virtual basketball using their eye gaze to select a virtual ball. Once the ball was selected the users were asked to click on any button on the Oculus Rift remote to grab it and propose the throwing force, this was visualized by an orange bar that surrounds the ball. When the users were satisfied with the force, they were instructed to release the button to fire the ball into the basket they were looking at. To increase the difficulty and reduce familiarity, several baskets in complex positions were presented to the users. A variety of feedback was used to increase the users’ engagement (e.g. sound effect, ambient sound, particle effects on every interaction, physics, additional items to target, pool with water properties such as buoyancy, moving vehicles) (see Fig. 2).

*Rationale* Cooling sensation was induced via dark, cold colours (e.g. green, blue, black) and strict edgy objects (squares, cubes, tetragons, rectangles). Based on the Multiple Resources Theory (Wickens 2008), a higher level of distraction can be achieved by multiple sensory signals, for this reason, we used multiple baskets, additional targets, moving background objects like birds, smoke from chimneys, and vehicles.

The physics of the ball require a mixture of skill and luck to make a score as the ball was affected by gravity. A scoring system was also used to increase users’ engagement and encouragement. This score was shown at all time and was animated to make the experience more frenetic and energized.

### Phase 2: system evaluation with induced pain in healthy population

#### Time to pain occurrence and tolerance

We measured the first time point at which pain was reported by the subject as an indication of pain occurrence. The data revealed that the occurrence of pain was faster during the documentary on visual impairments followed by the birthday celebration of a hedgehog. Interactive Virtual Environments were found to increase the time of pain occurrence by 50% (see Table 1). The above findings were further corroborated by the fact that the two interactive virtual environments followed by the birthday celebration of a hedgehog were able to double the exposure time to painful stimuli (see Table 1).

**Table 1:** Means and standard deviations of time to pain occurrence and tolerance for the four virtual environments

	Birthday celebration		Documentary		Puzzle		Basketball	
	*M*	SD	*M*	SD	*M*	SD	*M*	SD
Pain occurrence not reported	00:49	0.59	00:38	0.21	01:03	0.37	01:17	0.55
Maximal exposure reached	03:24	2.01	02:22	1.29	03:41	1.54	04:07	1.47

It worth mentioning, that during the three virtual environments (birthday celebration of a hedgehog, puzzle-based and basketball virtual experience) some participants did not report any pain at all. Those participants reached the maximal exposure of time, set to five minutes. The above findings were supported by the three virtual environments with slightly increased rates towards the interactive virtual environments (see Table 2).

**Table 2:** Frequencies of pain not reported and maximal exposure for the four virtual environment

	Birthday celebration	Documentary	Puzzle	Basketball
Pain occurrence not reported	2	0	3	4
Maximal exposure reached	9	1	10	9

#### Documentary on visual impairments reports

Based on the participants reports the documentary on visual impairments was characterized to be *“emotional” [Participant 6], “calming” [Participant 3], “with slow pacing” [Participant 3 and 15]* but also *“uncomfortable and boring” [Participant 1 and 8]. “However, I had to admit that it made me empathic. There were times that I wanted to close my eyes, and I may did it for a couple of seconds, it was like something was driving me to close my eyes to feel what that person was going through” [Participant 1].*

The participants also suggested that during the documentary they were more alerted to painful signals. Specifically, they quoted that: *“If I compare this virtual environment [referring to a documentary on visual impairments] to the rest [referring to the birthday celebration of a hedgehog, puzzle-based virtual environment, and basketball virtual experience] I can tell that I was feeling much more pain during this one” [Participant no3], “not only the pain incidences were stronger, but also the duration of each pain episode last for longer” [Participant no4]*.

Participant 10 suggested that the level of pain he was feeling was *“the same as with not having a VR headset on”* while participant 13 suggested that *“it was even more painful than without the VR”*. Also, it was reported that extra effort was needed *“to forget about the pain” [Participant 12]* and that the documentary on visual impairments made the participant *“breathe heavily” [Participant 8].* All the above might be explained by the fact that: *“During the documentary on visual impairments [the participant] was highly aware of the water, which removed the immersion” [Participant 11]* which resulted in *“not being able to tolerate the pain at all. I was feeling like the pain went up to 100%” [Participant 15].*

#### Birthday celebration of a hedgehog reports

Participants reports on the birthday celebration of a hedgehog were much more positive compared to the documentary on visual impairments. In particular, they characterized this virtual environment to be *“lovely” [Participant 1 and 15], “active” [Participant 3], “engrossed” [Participant 5], “fun” [Participant 4 and 6], “enjoyable” [Participant 7], “colourful and interesting” [Participant 14]*. The participants also reported that once they felt pain *“the pain plateaued and at the end didn’t get worse” [Participant 1].*

They also reported that *“even though the level of pain I felt during the experiment was similar to the baseline pain, however, I did notice some fluctuations” [Participant 3]* which worsened during the *“boring peaks of the film [referring to the virtual environment scenes]” [Participant 4].* The negative comments from the participants were further enhanced by the fact that participants felt that during the exposure they were *“getting used to it” [Participant 10],* which made the exposure *“boring” [Participant 9], “perhaps fun and effective only during the 1st time of viewing” [Participant 4],* and *“a good distraction only at the initial stages” [Participant 11]*. These statements are suggesting that long-term exposure to this virtual environment will be ineffective.

#### Puzzle-based experience reports

Participants characterized the puzzle-based virtual environment to be a *“beautiful environment” [Participant 1], “interactive” [Participant 2 and 3], “challenged” [Participant 1 and 4], “funny” [Participant 5], “entertained” [Participant 5, 8 and 9]* and *“fun” [Participant 6].* Participants reports regarding the feeling of pain were also positive suggesting that *“Pain didn’t seem to be so bad and was also tolerable after the first occurrence but to do so [to tolerate the pain] I had to be really focused” [Participant 1].*

The participants also suggested that the puzzle-based virtual environment made them able to extend their exposure in cold pressor even more than the maximal experimental time (five minutes): *“I felt that I could last even longer since I was not close to taking my hand out of the water” [Participant 3].*

Participants suggested that *“the pain did not feel as bad as in the birthday celebration and the documentary” [Participant 7]* and *“not only the pain was not that strong in comparison to the other sessions [referring to the birthday celebration of a hedgehog and documentary on visual impairments] but also the duration of the episodes was quite brief” [Participant 4].* As participants suggested the increased tolerance to pain stimuli might have resulted by the fact that they were *“much more concentrated” [Participant 6].* In particular, it was reported that being *“much more focused, made me able to… last even longer than the allowed time” [Participant 5].*

#### Basketball virtual experience reports

Similar positive results to the puzzle-based virtual environment were reported by the subjects during their exposure to basketball virtual experience. As aforementioned, the participants found the basketball virtual experience to be *“challenging” [Participant 1 and 8] “competitive” [Participant 4], “interactive” [Participant 2 and 3], “highly immersive” [Participant 5 and 13], “real” [Participant 9],* which *“held the attention to the best” [Participant 11], “fun” [Participant 5, 12 and 14] and “highly engaging” [Participant 15]*. From all four virtual environments, basketball virtual experience had the most tolerant responses to pain. In particular, the subjective reports were positive with the participants suggesting that *“it didn’t feel uncomfortable at all” [Participant 3].* The reported pain was much lower when compared to the other virtual environments (birthday celebration of a hedgehog, a documentary on visual impairments and puzzle-based) with most of the participants reporting that: *“It took me some time to realize that I was feeling some pain” [Participant 7], “I kept waiting for the pain to come, but it was taking some time and then it wasn’t too bad, it was also felt kind of different from the pain I was feeling during the other sessions [referring to the birthday celebration of a hedgehog, and the documentary on visual impairments]” [Participant 1].*

Some participants suggested that they *“felt some pain every now and then, but it was easily ignored” [Participant 10],* in other words, *“the pain existed but I was noticing it less” [Participant 13]* while some others suggested that they have *“totally forgotten about their hand, as if it wasn’t hurt at all” [Participant 12]* and that *“compared to the other sessions [referring to the birthday celebration of a hedgehog, and the documentary on visual impairments] zero pain was felt at the beginning which lasted for a significant amount of time” [Participant 7].* Interestingly there was one participant who suggested that during the VR exposure she felt zero pain but once the HMD was removed and her hand was not any longer into the cold pressor, the pain occurred *“I felt zero pain during the experiment, but once the experiment was over I felt some pain, around 40%” [Participant 6]*. However, *“overall it was a good distraction from pain, it made the process feel less painful” [Participant 11].*

The above reports were in accordance with participants’ ratings of the preferable virtual environment. Participants selected as their first choice the basketball virtual experience, followed by the puzzle-based virtual environment. The two interactive virtual environments were then followed by the birthday celebration of a hedgehog choice. All the participants rated the documentary on visual impairments as their last choice.

### Phase 3: deployment of the virtual reality system in clinical settings and system’s evaluation with burn-injured patients

**Fig. 3:**
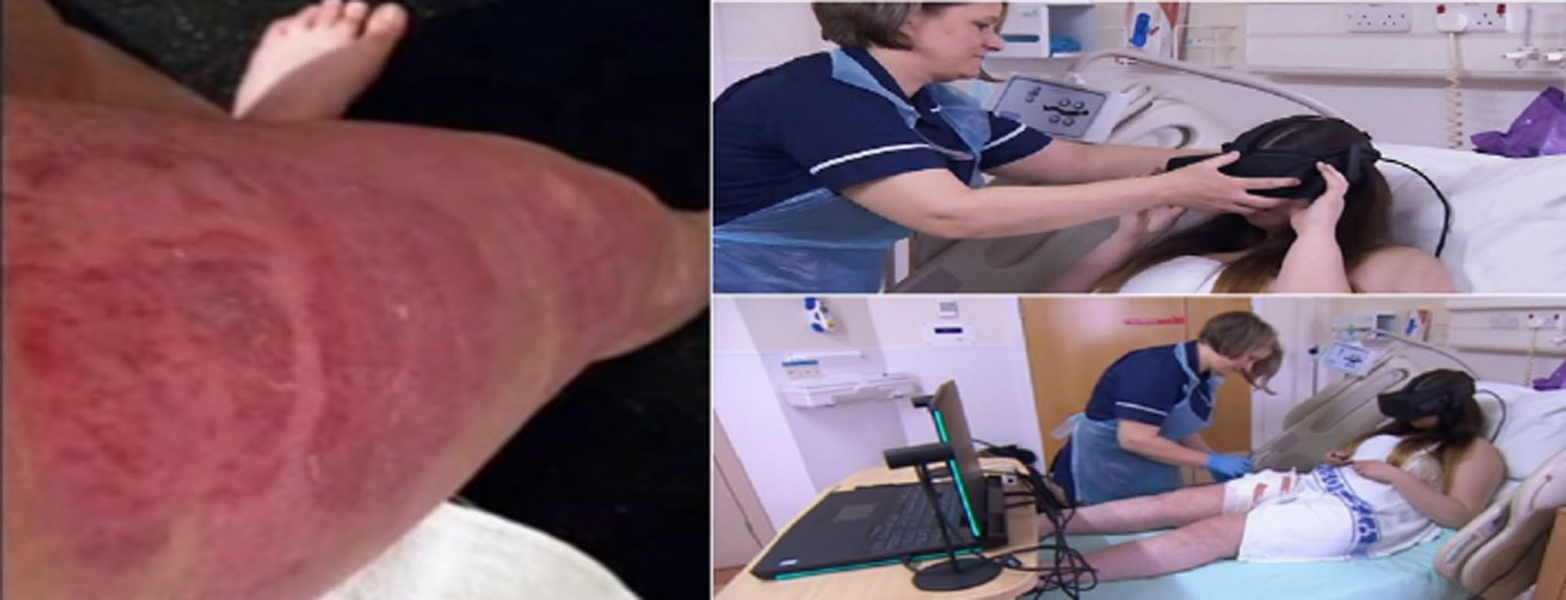
To the Left: the picture depicts the burn-injured body part. To the Right: the picture depicts the VR treatment

Based on the findings of experimental phase 2, the two interactive virtual environments (puzzle-based and basketball virtual experience) were selected for the clinical deployment of VR (Fig. 3). Our findings were complementary to the findings of induced pain via a cold pressor. Burn-injured patients were found to be able to tolerate perceived pain and increase the duration of the dressing changes. In particular, it was found that during the VR exposure the burn-injured patients were feeling significantly less pain compared to the regular wound debridement, dressing changes and wound cleaning where no VR intervention was used (Table 3). This results in an increased tolerance against pain, which allowed the nurses to remove more dead tissues from the wound. It was found that burn-injured patients were feeling significantly less anxious during the VR dressing changes compared to the non-VR (Table 3).

**Table 3:** *t* test results comparing the effect of VR on pain and anxiety ***p < 0.001; **p < 0.01; * < 0.05

	*n*	VR enhanced wound debridement		Regular wound debridement		*t*	df	*p*
		*M*	SD	*M*	SD			
Pain	5	44.00	17.10	52.50	15.54	6.75	4	0.007
Anxiety	5	36.00	33.61	56.25	31.51	3.36	4	0.044

Finally, it was quoted by the nursing staff that during the VR dressing changes less pharmacological analgesia was given to the burn-injured patients “*she was not in the need of any extra analgesia during, before or after the dressing changes. Normally, she would have asked for some [Nurse 3].* At the same time, the nurses reported that during the VR exposure they were able to spend more time on dressing changes and remove more surgical staples which resulted in reducing the overall duration of the healing process. This was contrary to the normal dressing changes processes where the burn-injured patient requested to terminate the dressing session after a shorter time or increase the pharmacological analgesia intake: *“He was a lot better with the VR on and I did pick quite a lot. Normally he does not allow the staff to do what we want to do because of the pain, whereas with the VR he allowed me to do that” [Nurse 1].*

#### Deployment challenges

*Challenge 1—The Anxiety of Change* Burn-Injured patients are dealing with painful therapeutic processes which support a bidirectional relationship between pain and anxiety (Li et al. 2011; Stoddard et al. 2006; Weinberg et al. 2000). Presence of pain and anxiety to burn-injured patients propose a significant challenge that needs to be addressed, especially when a new technology is proposed to the patients. During the deployment phase of VR in the clinical environment, two out of five patients were initially reluctant to engage in VR dressing changes therapy, but latter they were willing to try. As explained, the patients’ initial reluctance of using VR during their dressing changes comes from the unfamiliarity this technology presents. Specifically, it was quoted that *“I am stressed, I didn’t sleep well because I knew that today’s dressing changes will be very painful. I do not trust that this headset technology will help at all. I am dealing with chronic pain and relaxation or mindfulness technics are not any longer working for me, I do not expect this one to work either. I am just unwilling to try this, it makes me anxious and uncertain” [Patient 5].* Even though the patient’s reflection of VR technology was initially very negative we proposed to offer the patient a short but highly informative demonstration of VR technology. The demonstration was fast because of the patient’s intolerance. First, the equipment was given to the patient to familiarize him/herself with the material properties. Then a VR environment was projected on the computer screen to decrease the likelihood of any surprises. Finally, the same VR environment was presented to the patient through the HMD. During the whole process, a familiar nurse was sitting next to the patient to make her feel more comfortable. The same familiarization session was carried out with all five patients. Once the familiarization session was completed all five patients were less stressed and agreed in participating in VR dressing changes. It is worth mentioning that once the dressing changes were completed and the VR headset was off, the same patient commented that *“It was very useful, a really interesting experience. I would be glad to try it again. I felt like almost no pain, and I am proud to have achieved something through this” [Patient 5].* Similar attitudes were reported by the rest of the patients as well. One patient quoted: *“It was brilliant, I didn’t expect it to be so good. It took my mind off from pain and made me concentrate in the VR, this is a good fallback” [Patient 4].* While others commented: *“Pain arising from burn injuries is the worse pain I ever had, is even worse than childbirth, but I am telling you this technology can only be a good idea. It drags you off, it works to the best. I would like to always have this even if I had to pay for it” [Patient 3], “I was distracted, I wasn’t thinking about what they were doing. I am impressed. It [referring to the HMD] is worth its weight in gold” [Patient 1].*

*Challenge 2—Control and Independence* As opposed to normal care where the nursing staff are communicating the process to the patient (e.g. informing the patient about what exactly they are doing to the wound) to increase the patient’s control of the cleaning procedure and reduce anxiety, VR prevents the user’s visual access to the body, which reduce patients control of the session and as a result helps reduce anxiety triggers. Patient 1 quoted that *“control of the session was difficult. My suggestion is to increase control by reducing any kind of sounds incorporated to this [referring to the virtual environment], or it will be even better if the technology can detect speech and reduce the sounds during the talking. I would also like to be able to monitor the process, even though not being able to see what the nurse was doing reduced the pain I felt and so I allowed her [referring to the nurse] to do all the required steps. If I could have seen what she [referring to the nurse] was doing to my wounds I wouldn’t not let her to carry on”*.

*Challenge 3—Space and Equipment Restrictions* In contrast to deploying VR in other settings, we needed to understand the difficulties burn-injured patients encounter in physical moves and the hospital’s space restrictions. Some burn-injured patients are not able to get out of the bed and the only position comfortable to them is lying. As mentioned by patent 3, *“bed’s position made it hard for me to look around and I felt that the equipment might cause some sort of difficulty to the nurse”*. When we questioned whether the VR system caused difficulties to the nursing staff it was quoted that they found the process *“a lot better with the VR on, since it lessened the patient’s anxiety” [Nurse 1].* Also, they found the overall experience *“very positive and helpful, very good at distracting*” *[Nurse 2].*

On the other hand, and because the dressing change process took a substantial time some warmth was produced by the headset, which was spotted by the patients*: “I feel that there is a heat on the headset and this is making me nervous” [Patient 2] and “is a bit warm wearing it [referring to the headset]” [Patient 4]*. Finally, it should be noted that patients with burn injuries to the face and area that the headset is worn on, were excluded from the study.

*Challenge 4—Carry-overs of Pain* Even though positive results were reported during the dressing changes process, surprisingly once the dressing changes were over and the VR was removed from the burn-injured patients head, an increased pain occurred (*M* = 42.00, SD = 45.36) compared to the normal care process (*M* = 21.25, SD = 14.93). The pain was sharpened even more after two (*M* = 51.00, SD = 41.89) and four (*M* = 46.25, SD = 43.46) hours passed from the VR use, while this was not the case after two (*M* = 12.50, SD = 18.93) and four (*M* = 15.00, SD = 10.80) hours passed from the normal dressing changes, where pain faded. The above findings were further corroborated by the patients quotes who claimed that *“Afterwards it was so painful and so I kept thinking of it [referring to the VR experience] to take my mind off and it took it” [Patient 4], “two hours letter the dressing changes I reflected back on the VR and it had an effect of taking you away from the immediate trauma” [Patient 5]*.

#### Implications for design and successful deployment in clinical settings

*Use cartoons to increase user’s positive emotional responses* Based on the findings of the birthday celebration of a hedgehog virtual environment, we found that cartoons are an effective way to present virtual objects since that virtual environment was perceived to be a lovely environment by the users. Even though it didn’t prove to be as effective as the interactive virtual environments, however, it managed to increase user’s tolerance to pain. This is probably because cartoonish features can enhance tolerance to pain (Gold et al. 2006) even within clinical environments (Cohen et al. 1997; Lee et al. 2012). Previous studies claimed that the positive association of cartoonish features and less perceived pain is due to the fact that watching cartoons invoked happy childhood memories and improved mood (Bower 1981; Martin and Metha 1997; Matsangidou et al. 2019). Therefore, it is highly suggested for the virtual environments to incorporate animated, cartoonish elements.

*Attention should be given on personal interests* With respect to the negative findings produced by the documentary on visual impairments, we concluded that not all distractive virtual environments can decrease pain. Even though empathic reactions were generated and resulted in immersing the user into the virtual environment and even though visual cues were enhanced with blue colours to induce cooling sensations and tolerate pain (Candas and Dufour 2005; Durgin et al. 2007; Martini et at. 2013; Moseley and Arntz 2007), however, the results were negative. Participants reported to be bored and as a result, they were experiencing pain. Therefore, individual preferences must be taken into consideration. It is suggested for future studies to offer a set of different virtual environments to choose from to minimize the risk of unrelated context. These practices have been long used in the HCI community for designing successful VR content for individuals leaving with dementia (Hodge et al. 2018; Rose et al. 2018, 2019; Tabbaa et al. 2019) but not for burn-injured patients.

*Increase the interactivity to decrease the pain* Based on our findings an increased rate of interactivity can decrease the pain perception during the cold pressor experience or the painful dressing changes. A person’s attentional resources are limited and to cope with the incoming information, the person must select only the relevant to its aim and ignore the rest (Wickens 2008). Via VR, multi-sensory information is provided to the user, which divert the attentional resources away from the painful signal (Gold et al. 2007). Therefore, it is suggested that rehabilitation systems for painful procedures should be enhanced with increased interactivity to produce a multi-level of distraction by withdrawing the user’s attention from the painful sensory signal and place it on the successful task performance. It is also suggested, to enhance the system’s narration with unexpected turns and challenges to reduce familiarity and negative outcomes of carry-over effects.

*Familiarization with the equipment is a vital factor in burn-injured patients* As aforementioned, during the dressing changes treatment, anxiety due to pain commonly occurs (Li et al. 2011; Stoddard et al. 2006; Weinberg et al. 2000). High anxiety was reported by our patients, which resulted in an unwillingness to try VR technology. Our patients were exposed for the first time to VR and therefore, the unfamiliarity of this technology made the patients sceptical. Taking into consideration the burn-injured trauma, along with the hospitalization and the severe chronic pain, their unwillingness to try a new technology was not a surprise. To overcome this issue, we provided all patients with a familiarization session where VR was demonstrated to them as part of the informed consent procedure. Familiarizing themselves with the equipment made the patients less anxious and more willing to try the technology during their dressing changes. Therefore, it is suggested that a short demo or video footage of the proposed technology should offer to each patient before any intervention takes place, to reduce anxiety emerging from the fear of the unknown.

*Understand each patient's needs* Some patients are more comfortable when they can monitor or control the dressing changes process. VR produces some form of uncertainty which made the patient more anxious about the process because visual access to the body and the dressing changes process is prevented. However, not being able to observe the process increases the therapeutic outcomes and compared to the normal dressing changes, more surgical staples could potentially be removed resulting in more extensive debridement. Research suggests that a fake hand can be perceived as a real part of the body (Botvinick and Cohen 1998). Previous research in VR indicated that pain can not only be induced through a fake body part (Capelari et al. 2009) but also can be reduced if the appearance of the body part concealed visual cues that are related to pain, such as the redness of the skin (Hegedüs et al. 2014; Matsangidou et al. 2017a, b, 2019). Therefore, for those patients who would like to experience some form of control during their dressing changes, it is suggested to design a healthy-looking avatar and present the wounds in a way which will be unrelated to pain. For example, previously it was claimed that cartoonish elements can aid recovery from anxiety, therefore, wounds on the avatar skin might be presented as butterflies and each time the nursing staff successfully manage to remove a surgical staple, a butterfly will get free and fly around the patient.

*Consider the device portability* Choosing a technology that is portable and easy to set up to hospital environments is essential. During our study, the equipment was set up in the burn-injured patient's hospital treatment room. A speedy and easy setup of the equipment is a crucial factor to avoid patients experiencing any sort of discomfort. Oculus Rift HMD is connected to a PC via a wire, and to ensure that the patient was comfortable, and the wire would not affect the patient or interrupt negatively the nursing stuff job, we used the bed table to position the PC close to the patient. Disposable hygiene masks were used for each patient to reduce the risk of transmitting sweat, dirt, and germs to other patients. An alternative solution would have been to use wireless mobile VR HMD (e.g. Oculus Quest), which will decrease space restriction, but also will compromise the virtual environments’ quality and possibilities which have recently become available.

*Consider extending VR use after the dressing changes* VR has been proved to be a successful solution for managing pain during painful wound debridement, dressing changes, wound cleaning, and other medical procedures (Carrougher et al. 2009; Furness et al. 2019; Hoffman et al. 2014; Kipping et al. 2012; Maani et al. 2011; Schmitt et al. 2011). However, and to the best of our knowledge, this is the first study which examined the effects of VR on pain once those medical procedures are completed. Our findings suggested that intense pain which lasts for hours occurs to burn-injured patents when the VR is not any longer in use. This might be due to the nursing staff removed more dead tissues and surgical staples from the wound with a reduced dose of pharmacological analgesics, because of the effective VR analgesia. In line with our findings, a previous study suggested that VR can reduce significantly the need for pharmacological analgesics but haven’t tested the after-effect of this reduction (Christie et al. 2000). Therefore, we suggest future studies to examine the analgesic impact of offering extended VR exposure after the dressing changes.

## Conclusions, limitations and future directions

Burn-injured patients’ recovery requires dealing with painful therapeutic processes. Although these processes are fundamental for their recovery, by improving the functional outcomes and minimizing persistent disabilities, burn-injured patients often neglect to participate fully in their therapies (Richardson and Mustard 2009) due to the significant procedural pain (Ehde et al. 1998; Patterson and Sharar 2001). Several studies on burn-injured patients suggested that VR can be a suitable solution for managing the procedural pain (Carrougher et al. 2009; Furness et al. 2019; Hoffman et al. 2014; Kipping et al. 2012; Maani et al. 2011; Schmitt et al. 2011). This is because the attentional resources of the brain are limited and so when the subject is concentrated on a mental task (e.g. a game), the perceived pain is reduced (Melzack and Wall, 1965). VR has the ability to distract the subject’s attention from the signal of pain towards a complete distractive and entertain illusion (Li et al. 2011).

The purpose of this study was to explore whether VR is a feasible solution for burn-injured patients’ who are dealing with the painful therapeutic process, such as, wound debridement, dressing changes, wound cleaning, and therapeutic skin stretching within a hospital environment. Using qualitative and quantitative approaches we found that VR could be a successful solution for pain management in burn-injured patients when specific factors of the design are followed. In particular, our findings suggested that VR appeared to be very effective for this clinical population if the design includes features that respond to specific challenges, such as: (1) the use of cartoonish features to increase the patient’s positive emotions; (2) the use of a choice of content to match personal preference; (3) the use of interactive features to reduce the attentional resources to pain and increase the analgesia impact; (4) the portability of the equipment and the patient’s pre-exposure and familiarization; and (5) the extension of the VR use even after the therapeutic session is over.

In addition, this study described the process of how VR can be designed, tested, and deployed into a hospital environment based on the restrictions that burn-injured patients possess. The study was limited to a relatively small sample of burn-injured patients, constrained by time barriers of the clinical consent process and exclusion criteria. Nevertheless, the study contributes to the research in the design community by presenting a long-term experimental study for the effective design of a deployable VR system for burn-injured in-patients dealing with painful therapeutic processes within hospital units. In the future, studies should examine the use of VR for clinical rehabilitation in a large-scale sample. An additional limitation of the study was the absence of quantitative measures to verify the quoted findings related to the need for less pharmacological analgesia during the VR enhanced therapy. Even though Likert scales were used to document the reduction in patient’s pain and anxiety, we did not manage to get access to quantitative data regarding the pharmacological intake. It is therefore recommended for future studies to enhance data collection with data from the patient’s medical record and to compare the regular pharmacological intake during the therapeutic session with the pharmacological dosage which is given to the patient during the VR enhanced therapy. Finally, VR has been proved to be a successful solution for managing pain during painful wound debridement, dressing changes, wound cleaning, and other medical procedures but with a detrimental effect on pain once those medical procedures are completed. Our findings suggested that intense pain which lasts for hours occurs to burn-injured patents when the VR is no longer in use. Therefore, we suggest future studies to examine the analgesic impact of offering extended VR exposure after the dressing changes.

To conclude, this study contributes to the emerging body of research on the use of medical technology for burn-injured patients dealing with the painful therapeutic process in real-world clinical settings. The study presents the design process, the identification of the VR opportunities and the challenges we faced in the deployment of VR in the clinical settings. We believe this paper lays the foundations for the deployment of VR on a large scale in clinical environments and we can see a future where VR will be a part of the burn-injured patients’ medical therapies.

## Data Availability

Due to the nature of the study, the ethical approvals and the sensitivity of the data, we are not allowed to share the raw data.
